# Multidisciplinary team meetings: are all patients presented and does it impact quality of care and survival – a registry-based study

**DOI:** 10.1186/s12913-021-07022-x

**Published:** 2021-10-01

**Authors:** Quentin Rollet, Véronique Bouvier, Grégoire Moutel, Ludivine Launay, Anne-Laure Bignon, Karine Bouhier-Leporrier, Guy Launoy, Astrid Lièvre

**Affiliations:** 1 U1086 “ANTICIPE” INSERM-University of Caen Normandie, U1086 “Anticipe” – Centre François Baclesse, 3, Avenue du Général Harris, 14000 Caen, France; 2 Digestive Cancer Registry of Calvados, University Hospital of Caen, Avenue de la Côte de Nacre, 14000 Caen, France; 3 Espace Régional de Réflexion éthique, University Hospital of Caen, Avenue de la Côte de Nacre, 14000 Caen, France; 4 Department of Gastroenterology, University Hospital of Caen, Avenue de la Côte de Nacre, 14000 Caen, France; 5 Department of Gastroenterology, Rennes University Hospital 2 Rue Henri le Guilloux, 35000 Rennes, France; 6 U1242 “COSS” INSERM-University of Rennes, Centre Eugène Marquis, Rue de la Bataille Flandres Dunkerque, 35042 Rennes, France

**Keywords:** Disease management, Quality of health care, Health services accessibility, Health care ethics, Multidisciplinary team meeting

## Abstract

**Background:**

Multidisciplinary team meetings (MDTMs) are part of the standard cancer care process in many European countries. In France, they are a mandatory condition in the authorization system for cancer care administration, with the goal to ensure that all new patients diagnosed with cancer are presented in MDTMs.

**Aim:**

Identify the factors associated with non-presentation or unknown presentation in MDTMs, and study the impact of presentation in MDTMs on quality of care and survival in patients diagnosed with colorectal cancer (CRC).

**Methods:**

3999 CRC patients diagnosed between 2005 and 2014 in the area covered by the “Calvados Registry of Digestive Tumours” were included. Multivariate multinomial logistic regression was used to assess the factors associated with presentation in MDTMs. Univariate analyses were performed to study the impact of MDTMs on quality of care. Multivariate Cox model and the Log-Rank test were used to assess the impact of MDTMs on survival.

**Results:**

Non-presentation or unknown presentation in MDTMs were associated with higher age at diagnosis, dying within 3 months after diagnosis, unknown metastatic status, non-metastatic cancer and colon cancer. Non-presentation was associated with a diagnosis after 2010. Unknown presentation was associated with a diagnosis before 2007 and a longer travel time to the reference care centres. Presentation in MDTMs was associated with more chemotherapy administration for patients with metastatic cancer and more adjuvant chemotherapy for patients with stage III colon cancer. After excluding poor prognosis patients, lower survival was significantly associated with higher age at diagnosis, unknown metastatic status or metastatic cancer, presence of comorbidities, rectal cancer and non-presentation in MDTMs (HR = 1.5 [1.1–2.0], *p* < 0.001).

**Conclusions:**

Elderly and poor prognosis patients were less presented in MDTMs. Geriatric assessments before presentation in MDTMs were shown to improve care plan establishment. The 100% objective is not coherent if MDTMs are only to discuss diagnosis and curative cares. They could also be a place to discuss therapeutic limitations. MDTMs were associated with better treatment and longer survival. We must ensure that there is no inequity in presentation in MDTMs that could lead to a loss of chance for patients.

**Supplementary Information:**

The online version contains supplementary material available at 10.1186/s12913-021-07022-x.

## Introduction

Multidisciplinary team meetings (MDTMs) are periodical meetings between healthcare professionals with different medical expertise. The goals are to discuss patients’ diagnosis and conditions, and organize their treatment plan according to the most appropriate evidence-based protocols.

After being recommended by the Calman-Hine report in 1995 [1], in response to the inadequacy and uneven cancer care delivery perceived, the organisation of MDTMs was endorsed in the National Health Service Cancer Plan, published in 2000 in the United Kingdom. Since, it has been implemented in many western countries. In France, it was initiated through the launch of the “Plan Cancer” on the 24th of March 2003 [2]. One of the key measures of this plan was to ensure that 100% of new patients diagnosed with cancer were presented in MDTMs, in order to receive the most adequate and up-to-date treatment equally throughout the country. MDTMs are part of an ethical approach aimed at clarifying therapeutic objectives and questioning the proportionality of care, by giving a relevant opinion on all the therapeutic possibilities, and ensuring coherence between treatment plans and patients’ conditions.

Generalization of MDTMs has been truly effective since 2007, after publication of a decree stipulating that organisation of MDTMs was a mandatory condition for chemotherapy and external radiotherapy administration, and for cancer surgery practices [3].

With more than 43,000 colorectal cancer (CRC) cases diagnosed each year in France [4], it ranks third among the most common cancers in men and second in women. CRC management requires the knowledge and expertise of a wide range of healthcare specialists, making of MDTMs a central point in the care coordination of patients. A particularity of this cancer localisation is its decentralised care management, where diagnosis and surgery are not necessarily performed in expert health centres.

Since the national objective is to ensure that all new patients diagnosed with cancer are presented in MDTMs, it is important to study if this is the case. This is important to assess the impact and feasibility of public health policies, to improve practices, and to ensure whether patients’ fundamental rights are respected.

The aim of this study was to evaluate the implementation of MDTMs as part of CRC care management in the area covered by the “Calvados Registry of Digestive Tumours”, by identifying the factors associated with non-presentation or unknown presentation in MDTMs, and studying the impact of presentation in MDTMs on quality of care and survival.

## Materials and methods

### Population and variables

Thanks to the Calvados Registry of Digestive Tumours, all 4032 patients diagnosed with CRC in Calvados (Normandy, France) between 2005 and 2014 (C18-C19-C20; ICDO-3) were included in this study. This period was chosen to ensure sufficient follow-up and survival data for patients in 2019*.* Data are actively collected by the cancer registry staff from a university hospital, a regional comprehensive cancer centre, 12 public or private general hospitals (two with radiotherapy facilities), and 4 pathology laboratories. The databases are declared to the National Commission on Information Technology and Civil Liberties (CNIL) and the cancer registry is part of the French network of cancer registries (FRANCIM). Data quality and completeness are assessed by the CER (Comité d’Évaluation des Registres) and by the International Agency for Research on Cancer (IARC). Patient’s sex, place of residence, clinical and pathological stages according to the TNM 7, date of diagnosis, tumour site, Charlson’s comorbidity index (CCI), vital status, treatment and presentation in MDTMs were extracted from the registry. In Calvados, patients with CRC are presented in MDTMs specific to digestive cancers. How they are organised has been changed over time, with a trend towards centralisation and collaboration between care centres. However, these changes are frequent, centre dependent, and not routinely recorded. Patients’ cases were considered presented or not in MDTMs if their medical records clearly stated the information. The status of patients who had not met this requirement were considered “unknown”. In this study, we considered patients’ presentation in MDTMs after their first contact with the health care system concerning their CRC diagnosis.

Patients were geolocalized and allocated to their residential IRIS (*Îlots regroupés pour l’Information Statistique*). Each of these IRIS correspond to a European Deprivation Index (EDI) score [5], used in quintiles in this study (the first one representing the least and the fifth the most deprived). We also computed travel time between patients’ places of residence and the oncology reference hospital centres (University hospital and regional comprehensive cancer centre), using Navstreets® V14.0 data (provided by HERE and Esri France) and the Network Analyst extension of ArcGIS© PRO software.

Finally, we included periods. The first corresponded to patients diagnosed in 2005 and 2006, when presentation in MDTMs was strongly recommended (the 100% objective was set up in 2003). The second corresponded to patients diagnosed between 2007 and 2010, after that organisation of MDTMs became a mandatory condition to deliver cancer care and while the registry was adjusting to better collect information on presentation in MDTMs. The third corresponded to patients diagnosed after 2010, when organisation of MDTMs was supposedly well implemented and presentation in MDTMs well registered in the registry.

After excluding patients whose addresses could not be geolocalized (*n* = 33), the final population was 3999 patients. Metastatic status, CCI and presentation in MDTMs all presented missing data. In this study, we chose to keep the « unknown » categories, because they reflect the quality of data recording in patients’ medical records, either by medical or registry staff.

### Analysis plan

In the first part of this study, we used multinomial logistic regression (a method that generalises logistic regression to multiclass problems [6]) to assess the factors associated with the non-presentation and unknown presentation in MDTMs.

In the second part, we assessed whether presentation in MDTMs was associated with the quality of care, by studying three criteria:

1. The proportion of patients with metastatic CRC who received chemotherapy;
2. The proportion of patients with a pathological stage III colon cancer who received adjuvant chemotherapy;
3. The proportion of patients with clinical locally advanced rectal cancer who received neoadjuvant chemoradiotherapy;

These treatments are considered therapeutic standards according to French [7–9] and European guidelines [10–12]. Chi-squared tests were performed to compare each criterion between the three MDTM groups (presented, not presented, unknown) when all expected values were equal to or higher than 5, otherwise, Fisher’s exact tests were performed. Due to the small sample sizes, multivariate models were not carried out.

In the last part of this study, we used Cox model and the Log-Rank test to compare survival between the patients presented in MDTMs and those who were not. We excluded patients for whom presentation in MDTMs was unknown because of the probable mixture of those presented and not presented that can’t be interpreted. Analysis was repeated excluding patients dying within 3 months after CRC diagnosis, as this delay allows the identification of patients of particularly poor prognosis (widespread and incurable metastatic disease, major comorbidities and poor general condition), to whom supportive care alone, rather than active therapy, is mostly recommended. Moreover, it is usually admitted by oncologists that chemotherapy is not proposed if the expected survival is lower than 3 months [13].

This article was written following the STROBE guidelines [14].

## Results

Characteristics of the population by presentation in MDTMs are described in Table 1.

**Table 1:** Characteristics of the population

Total	*n* = 3999	Presentation in MDTMs		
	n (%)	Yes	No	Unknown
		3298 (83)	174 (4)	527 (13)
Sex				
Male	2203 (55)	1847 (84)	80 (4)	276 (12)
Female	1796 (45)	1451 (81)	94 (5)	251 (14)
Age at diagnosis (years)				
≤50	228 (6)	213 (94)	3 (1)	12 (5)
[51–65]	1040 (26)	936 (90)	14 (1)	90 (9)
[66–75]	1092 (27)	918 (84)	28 (3)	146 (13)
[76–80]	478 (12)	395 (83)	20 (4)	63 (13)
> 80	1161 (29)	836 (72)	109 (9)	216 (19)
Death within 3 months after diagnosis				
No	3638 (91)	3105 (85.4)	89 (2.5)	444 (12.2)
Yes	361 (9)	193 (53.5)	85 (23.6)	83 (23)
Tumour localisation				
Colon	2829 (71)	2260 (80)	142 (5)	427 (15)
Rectal	1170 (29)	1038 (89)	32 (3)	100 (8)
Stage at diagnosis				
I	779 (20)	619 (79)	29 (4)	131 (17)
II	920 (23)	777 (85)	29 (3)	114 (12)
III	808 (20)	726 (90)	15 (2)	67 (8)
IV	1090 (27)	941 (86)	43 (4)	106 (10)
Non-operated and non-metastatic	223 (6)	141 (63)	38 (17)	44 (20)
Unknown	179 (4)	94 (53)	20 (11)	65 (36)
Metastasis				
Yes	1090 (27)	941 (86)	43 (4)	106 (10)
No	2828 (71)	2329 (82)	115 (4)	384 (14)
Unknown	81 (2)	28 (34)	16 (20)	37 (46)
Charlson Comorbidity Index				
0	1907 (48)	1678 (88)	49 (3)	180 (9)
1	850 (21)	702 (83)	52 (6)	96 (11)
> 1	858 (21)	670 (78)	61 (7)	127 (15)
Unknown	384 (10)	248 (65)	12 (3)	124 (32)
Travel time quintiles (min)				
Q1 [3–10]	800 (20)	704 (88)	33 (4)	63 (8)
Q2 [11–17]	800 (20)	686 (86)	22 (3)	92 (11)
Q3 [18–28]	800 (20)	668 (83)	39 (5)	93 (12)
Q4 [29–39]	800 (20)	640 (80)	44 (5)	116 (15)
Q5 [40–75]	799 (20)	600 (75)	36 (5)	163 (20)
European Deprivation Index quintiles				
Q1 (less deprived)	800 (20)	676 (84)	30 (4)	94 (12)
Q2	802 (20)	668 (83)	32 (4)	102 (13)
Q3	799 (20)	658 (82)	44 (6)	97 (12)
Q4	808 (20)	652 (81)	33 (4)	123 (15)
Q5 (most deprived)	790 (20)	644 (82)	35 (4)	111 (14)
Period				
[2005–2006]	752 (19)	500 (66)	12 (2)	240 (32)
[2007–2009]	1137 (28)	940 (83)	23 (2)	174 (15)
[2010–1014]	2110 (53)	1858 (88)	139 (7)	113 (5)

Overall, 3298 (83%) patients were presented in MDTMs, 174 (4%) were not presented, and presentation in MDTMs was unknown for 527 (13%) patients.

### Factors associated with no or unknown presentation in MDTMs

All results for univariate and multivariate analyses are presented in Table 2.

**Table 2:** Factors associated with non-presentation or unknown presentation in MDTMs

	Univariate - OR (CI)		Multivariate - OR (CI)	
MDTM status	No	Unknown	No	Unknown
Sex	***p = 0.01***	*p = 0.12*	*p = 0.34*	*p = 0.97*
Male	1	1	1	1
Female	1.5 (1.1–2.0)	1.2 (1.0–1.4)	1.2 (0.8–1.7)	1 (0.8–1.2)
Age at diagnosis (years)	***p < 0.001***	***p < 0.001***	***p < 0.001***	***p < 0.001***
≤50	1	1	1	1
[51–65]	1.1 (0.3–3.7)	1.7 (0.9–3.2)	1 (0.3–3.6)	1.7 (0.9–3.3)
[66–75]	2.2 (0.7–7.2)	2.8 (1.5–5.2)	1.8 (0.5–6.3)	2.5 (1.3–4.7)
[76–80]	3.6 (1.1–12.2)	2.8 (1.5–5.4)	2.3 (0.6–8.3)	2.5 (1.2–4.8)
> 80	9.3 (2.9–29.4)	4.6 (2.5–8.4)	3.8 (1.1–13)	3.8 (2.0–7.2)
Death within 3 months after diagnosis	***p < 0.001***	***p < 0.001***	***p < 0.001***	***p < 0.001***
No	1	1	1	1
Yes	15.4 (11–21.4)	3.0 (2.3–4.0)	12.1 (8.2–18)	2.8 (2–3.9)
Tumour localisation	***p < 0.001***	***p < 0.001***	***p = 0.02***	***p < 0.001***
Colon	1	1	1	1
Rectal	0.5 (0.3–0.7)	0.5 (0.4–0.6)	0.6 (0.4–0.9)	0.5 (0.4–0.6)
Metastasis	***p < 0.001***	***p < 0.001***	***p < 0.001***	***p < 0.001***
Yes	0.9 (0.7–1.3)	0.7 (0.5–0.9)	0.5 (0.3–0.8)	0.5 (0.4–0.7)
No	1	1	1	1
Unknown	11.6 (6.1–22)	8 (4.9–13.3)	4.5 (2–10)	4 (2.2–7.1)
Charlson Comorbidity Index	***p < 0.001***	***p < 0.001***	*p = 0.08*	***p < 0.001***
0	1	1	1	1
1	2.5 (1.7–3.8)	1.3 (1.0–1.7)	1.7 (1.1–2.6)	1.1 (0.8–1.5)
> 1	3.1 (2.1–4.6)	1.8 (1.4–2.3)	1.7 (1.1–2.6)	1.3 (1.0–1.7)
Unknown	1.7 (0.9–3.2)	4.7 (3.6–6.1)	1.5 (0.8–3.2)	2.6 (2.0–3.6)
Travel time quintiles (min)	*p = 0.05*	***p < 0.001***	*p = 0.12*	***p < 0.001***
Q1 [3–10]	1	1	1	1
Q2 [11–17]	0.7 (0.4–1.2)	1.5 (1.1–2.1)	0.7 (0.4–1.2)	1.8 (1.3–2.7)
Q3 [18–28]	1.3 (0.8–2.0)	1.6 (1.1–2.2)	1.2 (0.7–2.1)	1.6 (1.1–2.3)
Q4 [29–39]	1.5 (0.9–2.3)	2.0 (1.5–2.8)	1.4 (0.8–2.3)	2.4 (1.7–3.5)
Q5 [40–75]	1.3 (0.8–2.1)	3.0 (2.2–4.1)	1.3 (0.8–2.3)	3.5 (2.5–4.9)
European Deprivation Index quintiles	*p = 0.47*	*p = 0.22*	*p = 0.55*	*p = 0.72*
Q1 (less deprived)	1	1	1	1
Q2	1.1 (0.7–1.8)	1.1 (0.8–1.5)	0.9 (0.5–1.6)	1.0 (0.7–1.4)
Q3	1.5 (0.9–2.4)	1.1 (0.8–1.4)	1.1 (0.6–1.8)	0.8 (0.6–1.2)
Q4	1.1 (0.7–1.9)	1.4 (1.0–1.8)	0.7 (0.4–1.3)	1.0 (0.7–1.4)
Q5 (most deprived)	1.2 (0.7–2.0)	1.2 (0.9–1.7)	0.8 (0.4–1.3)	0.9 (0.6–1.3)
Period	***p < 0.001***	***p < 0.001***	***p < 0.001***	***p < 0.001***
[2005–2006]	1	1	1	1
[2007–2009]	1.0 (0.5–2.1)	0.4 (0.3–0.5)	0.8 (0.4–1.8)	0.3 (0.3–0.4)
[2010–1014]	3.1 (1.7–5.7)	0.1 (0.1–0.2]	3.0 (1.6–5.6)	0.1 (0.1–0.2]

In multivariate analysis, no presentation in MDTMs was significantly associated with higher age at diagnosis (OR_> 80_ = 3.8 [1.1–13], compared with < 50 years; *p* < 0.001), dying within 3 months after diagnosis (OR = 12.1 [8.2–18.0]; *p* < 0.001), unknown metastatic status (OR = 4.5 [2.0–10.0]) or non-metastatic cancer (OR_M+_ = 0.5 [0.3–0.8; p < 0.001), colon cancer (OR_RECTAL_ = 0.6 [0.4–0.9]; p < 0.001) and a diagnosis after 2010 (OR = 3.0 [1.6–5.6]; *p* < 0.001). Female sex and CCI were associated in univariate, but not in multivariate analyses. Travel time and deprivation had no effects.

In multivariate analysis, unknown presentation in MDTMs was associated with higher age at diagnosis (OR_66–75_ = 2.5 [1.3–4.7], OR_76–80_ = 2.5 [1.2–4.8]; OR_> 80_ = 3.8 [2.0–7.2]; *p* < 0.001), dying within 3 months after diagnosis (OR = 2.8 [2.0–3.9]; p < 0.001), unknown metastatic status (OR = 4.0 [2.2–7.1]) or non-metastatic cancer (OR_M+_ = 0.5 [0.4–0.7; *p* < 0.001), unknown CCI (OR = 2.6 [2.0–3.6]) or CCI > 1 (OR = 1,3 [1.0–1.7]; *p* < 0.001), longer travel time to the reference care centres (OR_Q2_ = 1.8 [1.3–2.7]; OR_Q3_ = 1.6 [1.1–2.3]; OR_Q4_ = 2.4 [1.7–3.5]; OR_Q5_ = 3.5 [2.5–4.9], compared with Q1; p < 0.001), colon cancer (OR_RECTAL_ = 0.5 [0.4–0.7]; p < 0.001) and a diagnosis before 2007 (OR_2007–2009_ = 0.3 [0.3–0.4]; OR_2010–2014_ = 0.1 [0.1–0.2]; *p* < 0.001). Factors associated with unknown presentation in MDTMs were the same in univariate and multivariate analyses. Sex and deprivation had no effects.

### MDTMs and quality of care

Among patients with metastases (Table 3), 65.7% received chemotherapy (62.5% for colon cancer, 75.2% for rectal cancer; *p* < 0.001). Chemotherapy was administered for 71.5% of patients presented in MDTMs, 9.3% of patients not presented in MDTMs and 36.8% of patients with unknown presentation in MDTMs (*p* < 0.001). These results were found for both metastatic colon cancer (respectively 68.9, 8.1, 35.6%; p < 0.001) and metastatic rectal cancer (78.6, 16.7, 43.8%; p < 0.001). Other factors associated with chemotherapy administration are described in ﻿S1.

**Table 3:** Impact of MDTMs on quality of care

	Overall		Colon		Rectum	
**Metastatic**	*n* = 1090		*n* = 816		*n* = 274	
Chemotherapy	*No*	*Yes*	*No*	*Yes*	*No*	*Yes*
	374 (34)	716 (66)	306 (38)	510 (62)	68 (25)	206 (75)
Presentation in MDTMs		***p < 0.001***		***p < 0.001***		***p < 0.001***^***a***^
Yes	268 (28)	673 (72)	214 (31)	475 (69)	54 (21)	198 (79)
No	39 (91)	4 (9)	34 (92)	3 (8)	5 (83)	1 (17)
Unknown	67 (63)	39 (37)	58 (64)	32 (36)	9 (56)	7 (44)
**Non-metastatic N+**	*n* = 806		*n* = 608		*n* = 198	
Adjuvant chemotherapy	*No*	*Yes*	*No*	*Yes*	*No*	*Yes*
	252 (31)	554 (69)	220 (36)	388 (64)	32 (16)	166 (84)
Presentation in MDTMs		***p < 0.001***		***p < 0.001***		*p* = 1^a^
Yes	209 (29)	515 (71)	178 (33)	359 (67)	31 (17)	156 (83)
No	15 (100)	0	15 (100)	0	na	na
Unknown	28 (42)	39 (55)	27 (48)	29 (52)	1 (9)	11 (91)
**Locally advanced**					*n* = 259	
Pre-operative chemoradiotherapy					*No*	*Yes*
					70 (27)	189 (73)
Presentation in MDTMs						p = 0.5^a^
Yes					66 (27)	182 (73)
No					na	na
Unknown					4 (36)	7 (64)

^a^ exact Fisher test

Among patients with stage III cancer, 68.7% received adjuvant chemotherapy (63.8% for colon cancer, 83.8% for rectal cancer; *p* < 0.001). MDTMs were not associated with adjuvant chemotherapy administration for patients with rectal cancer. For colon cancer, adjuvant chemotherapy was administered for 66.9% of the patients presented in MDTMs, none of the patients not presented in MDTMs and 51.8% of patients with unknown presentation in MDTMs (p < 0.001). Other factors associated with adjuvant chemotherapy administration are described in S2.

Among patients with locally advanced rectal cancer, 72.6% received preoperative chemoradiotherapy. MDTMs were not associated with preoperative chemoradiotherapy administration. Only 11 patients (4%) had an unknown presentation in MDTMs, all others were discussed. Other factors associated with preoperative chemoradiotherapy administration are described in S3.

### MDTMs and survival

All results for univariate and multivariate analyses are presented in Table 4.

**Table 4:** Impact of MDTMs on survival

	Overall survival - HR (CI) *n* = 3472		Excluding patients dying within 3 months after diagnosis - HR (CI) n = 3194	
	Univariate	Multivariate	Univariate	Multivariate
Presentation in MDTMs	***p < 0.001***	***p < 0.001***	***p < 0.001***	***p = 0.005***
Yes	1	1	1	1
No	3.5 (3.0–4.2)	2.8 (2.4–3.4)	1.7 (1.3–2.3)	1.5 (1.1–2.0)
Sex	*p = 0.16*	*p = 0.16*	*p = 0.63*	*p = 0.14*
Male	1	1	1	1
Female	1.1 (1.0–1.2)	0.9 (0.9–1.0)	1.0 (0.9–1.1)	0.9 (0.8–1.0)
Age at diagnosis (year)	***p < 0.001***	***p < 0.001***	***p < 0.001***	***p < 0.001***
≤50	1	1	1	1
[51–65]	1.0 (0.8–1.2)	1.1 (0.8–1.3)	1.0 (0.8–1.2)	1.1 (0.8–1.3)
[66–75]	1.3 (1.1–1.7)	1.5 (1.2–1.9)	1.3 (1.0–1.6)	1.5 (1.2–1.9)
[76–80]	1.9 (1.5–2.5)	2.3 (1.8–2.9)	1.9 (1.5–2.4)	2.3 (1.8–3.0)
> 80	3.2 (2.5–3.9)	3.9 (3.1–4.8)	2.9 (2.3–3.6)	3.9 (3.1–4.9)
Tumour localisation	*p = 0.17*	***p = 0.005***	*p = 0.95*	***p < 0.001***
Colon	1	1	1	1
Rectal	0.9 (0.9–1.0)	1.2 (1.0–1.3)	1.0 (0.9–1.1)	1.2 (1.1–1.3)
Metastasis	***p < 0.001***	***p < 0.001***	***p < 0.001***	***p < 0.001***
Yes	4.3 (3.9–4.7)	5.5 (5.0–6.0)	4.4 (4.0–4.8)	5.7 (5.1–6.3)
No	1	1	1	1
Unknown	5.7 (4.2–7.8)	2.8 (2.1–3.9)	3.9 (2.5–5.8)	2.4 (1.6–3.7)
Charlson Comorbidity Index	***p < 0.001***	***p < 0.001***	***p < 0.001***	***p < 0.001***
0	1	1	1	1
1	1.5 (1.3–1.7)	1.3 (1.1–1.4)	1.5 (1.3–1.6)	1.3 (1.1–1.4)
> 1	1.9 (1.7–2.2)	1.7 (1.6–1.9)	1.9 (1.7–2.1)	1.8 (1.6–2.0)
Unknown	1.1 (0.9–1.3)	1.0 (0.9–1.2)	1.0 (0.8–1.2)	1 .0 (0.8–1.2)
Travel time quintiles (min)	*p = 0.60*	*p = 0.66*	*p = 0.82*	*p = 0.74*
Q1 [3–10]	1	1	1	1
Q2 [11–17]	1.0 (0.8–1.1)	1.0 (0.9–1.2)	1.0 (0.9–1.1)	1.1 (0.9–1.2)
Q3 [18–28]	1.0 (0.9–1.2)	1.0 (0.9–1.2)	1.0 (0.9–1.2)	1.0 (0.9–1.2)
Q4 [29–39]	1.1 (0.9–1.2)	1.1 (0.9–1.2)	1.1 (0.9–1.2)	1.1 (0.9–1.2)
Q5 [40–75]	1.0 (0.9–1.2)	1.0 (0.8–1.1)	1.1 (0.9–1.2)	1.0 (0.8–1.1)
European Deprivation Index quintiles	***p < 0.001***	*p = 0.27*	***p < 0.001***	*p = 0.14*
Q1 (less deprived)	1	1	1	1
Q2	1.0 (0.9–1.2)	1.0 (0.8–1.1)	1.0 (0.9–1.2)	1.0 (0.9–1.2)
Q3	1.1 (1.0–1.3)	1.0 (0.8–1.1)	1.1 (0.9–1.2)	1.0 (0.8–1.1)
Q4	1.3 (1.1–1.5)	1.1 (0.9–1.3)	1.3 (1.1–1.5)	1.1 (1.0–1.3)
Q5 (most deprived)	1.3 (1.1–1.4)	1.1 (0.9–1.3)	1.3 (1.1–1.5)	1.1 (1.0–1.3)

#### All population

In multivariate analysis, lower survival was significantly associated with higher age at diagnosis (HR_66–75_ = 1.5 [1.2–1.9], HR_76–80_ = 2.3 [1.8–2.9], HR_> 80_ = 3.9 [3.1–4.8], compared with < 50 years; *p* < 0.001), unknown metastatic status (HR = 2.8 [2.1–3.9]) or metastatic cancer (HR = 5.5 [5.0–6.0]; p < 0.001), a CCI ≥ 1 (HR_CCI = 1_ = 1.3 [1.1–1.4], HR_CCI > 1_ = 1.7 [1.6–1.9]; *p* < 0.001), rectal cancer (HR = 1.2 [1.0–1.3]; *p* = 0.005) and no presentation in MDTMs (HR = 2.8 [2.4–3.4]; *p* < 0.001). Higher deprivation was associated in univariate, but not in multivariate analyses. Sex and travel time had no effects.

#### Excluding patients who died within 3 months after diagnosis (*n* = 3194)

In multivariate analysis, lower survival was significantly associated with higher age at diagnosis (HR_66–75_ = 1.5 [1.2–1.9], HR_76–80_ = 2.3 [1.8–3.0], HR_> 80_ = 3.9 [3.1–4.9], compared with < 50 years; *p* < 0.001), unknown metastatic status (HR = 2.4 [1.6–3.7]) or metastatic cancer (HR = 5.7 [5.1–6.3]; p < 0.001), a CCI ≥ 1 (HR_CCI = 1_ = 1.3 [1.1–1.4], HR_CCI > 1_ = 1.8 [1.6–2.0]; p < 0.001), rectal cancer (HR = 1.2 [1.1–1.3], *p* = 0.005) and no presentation in MDTMs (HR = 1.5 [1.1–2.0]; p < 0.001). Higher deprivation was associated in univariate, but not in multivariate analyses. Sex and travel time had no effects.

## Discussion

In this large cohort of 3999 patients, there was an increased rate of patients presented in MDTMs over time, from 66% before it was made a mandatory condition to deliver cancer care (2005–2006) to 88% in more recent years (2010–2014). This last result is comparable to the national level, with 88% of patients with digestive system cancer presented in MDTMs in 2016 [15]. These rates were slightly lower to those found for patients with CRC in the Netherlands (93% in 2015 and 2016) [16] or for patients with rectal cancer in Belgium (91% in 2011) [17]. Data recording in the medical records/registry has improved, with lower rates of patients with unknown presentation in MDTMs, from 32% before 2007 to 5% after 2010. Nowadays, optimal care management for patients with CRC needs the consideration of clinical, biological, pathological, molecular and imaging parameters, and MDTMs are a great opportunity to bring together experts from these different fields. Neoadjuvant chemoradiotherapy administration for patients with non-metastatic rectal cancer, chemotherapy administration for some patients with stage II CRC, surgery of metastasis, multi-line chemotherapy strategy, relevance of chemotherapy and the most suitable regimen in elderly are some examples of topics needing discussion in MDTMs.

Some patients are not presented in MDTMs. This is particularly the case for poor prognosis patients (dying within 3 months after CRC diagnosis). We could hypothesize that patients’ conditions at diagnosis were already beyond any therapeutic solutions, leading the care provider to shift to palliative care. These patients explained most of the overall survival differences between those discussed or not in MDTMs. However, survival disparities were also observed between patients surviving at least 3 months, independently of age, comorbidities, metastasis and tumour localisation. This study adds strength to the evidence that MDTMs improve patients’ survival [18]. If the goal of MDTMs is only to discuss diagnosis and curative care, the 100% objective is not coherent. These meetings could also be a place to discuss therapeutic limitations or abstentions. Collective decisions resulting from discussion between experts in different fields are also needed before proposing supportive care, to ensure that care limitation is the best solution and that optimal palliative care is given. Leonetti’s law, about end-of-life and care limitation rights encourages such collegiality [19]. The benefits of integrating palliative care professionals in MDTMs have already been reported. They included more alteration from curative to palliative treatment [20–22] (or vice versa [22]), earlier referral to palliative care, decreased odds of dying at the hospital and of receiving chemotherapy in last 14 days of life [23], and more discussion about patients’ end-of-life preferences [24].

Elderly patients are also less presented in MDTMs. Age alone should not be a marker of disparity. In breast cancer, a study showed that 31% of patients considered fit after geriatric assessment had not received the appropriate adjuvant treatment [25]. Billon et al. [26] also showed a relation between age-adapted treatment and the use of geriatric-specific variables, G8 scores and comprehensive geriatric assessments in MDTMs. Whether or not a patient is fit to undergo a curative treatment, MDTMs could be useful in determining which care management would be most helpful, considering the results of geriatric assessment and patient’s frailty status. Geriatric oncology evaluations need to be ensured for the elderly, and these evaluations should be discussed in MDTMs.

Patients without metastasis were less presented in MDTMs, probably because treatment for localised forms of CRC are well standardized. Patients with rectal cancer were more presented in MDTMs, probably because rectal cancer treatment, notably for localised stages, often need discussion between oncologists, radiotherapists and surgeons, as well as a systematic cross checking by a digestive radiologist. In this study, all patients with locally advanced rectal cancer were presented in MDTMs (when presentation in MDTMs was known).

Longer travel time to the reference care centres was associated with more unknown presentation in MDTMs. Other studies on registry data showed poorer care management and survival for patients furthest from reference care centres [27, 28]. Even if, in this study, travel time was not associated with poorer survival, vigilance is needed to ensure health care equity. In addition, other factors associated with unknown presentation in MDTMs were mostly the same factors associated with non-presentation. Better evaluation of MDTMs and of their consequences needs accurate information for all patients. Finally, deprivation was not associated with presentation in MDTMs, despite being known to impact incidence and prognosis. To our knowledge, there is a lack of literature on the impact of social status on presentation in MDTMs. It seems that social equity was insured in this population regarding presentation in MDTMs.

Our results showed an association between MDTMs and quality of care. Patients with stage III or IV colon cancer presented in MDTMs were more often treated with chemotherapy than those not presented, and the same results were observed for patients with stage IV rectal cancer. In a systematic review of the impact of MDTMs [18], more than 10% of patients had diagnostic changes in 5 out of 9 studies and alteration in care management in 7 out of 13 studies after MDTMs. Appropriate, more precise and complete staging as well as neoadjuvant/adjuvant treatment were also associated with presentation in MDTMs. Weak evidence of patient’s referral to other disciplines after presentation in MDTMs were also highlighted. The main question is to know if the absence of discussion in MDTMs could be a loss of chance for patients. We must ensure that there is no inequity in care or therapeutic orientations for patients that could lead to inadequate treatment and poorer survival. Trying to understand the reasons for which a 100% objective is not achievable/achieved is thus essential.

MDTMs are also a place to discuss with professionals having no affective relation with the patient, ensuring regular discussions between professionals thereby improving teamwork [29] and communication [30]. They are important for initial training of students and continuing education of practitioners [30, 31]. Finally, they could also improve recruitment in clinical trials and homogenize practices [32]. However, for the medical staff, MDTMs are time-consuming. Some logistic difficulties need to be addressed in order to fully and efficiently implement MDTMs, mainly because of physician time constraints, but also administrative, informatic, registering and medical demography issues, as well as the low valorisation of this work. Public authorities need to be aware of these issues to avoid a decrease in quality of these practices.

Registry data are useful to evaluate the quantitative aspects of the presentation in MDTMs. Nearly all patients diagnosed between 2005 and 2014 were included in this study, and the registry contains very accurate data on patients’ conditions since diagnosis, on their treatment and their vital status, wherever they have been treated in *Calvados*. Geolocalization of patients makes it possible to enrich these data with patients’ socioeconomic and geographic information. Accounting for all these variables is important in understanding how each characteristic could influence patients’ outcomes.

Nevertheless, the registry lacks information on MDTMs duration, the professionals participating and the decisions taken. In addition, it covers only one area in France, and our results cannot be extrapolated to the entire country. More and larger studies are needed to understand why some patients are not presented in MDTMs as well as the effects of MDTMs on patients’ outcomes. It would also be interesting to evaluate if MDTMs are integrated equally in all health centres throughout the territory, leading to the same improvements in patients’ outcomes. Patients’ satisfaction and the impact of MDTMs on their quality of life are also relevant indicators needing to be studied.

## Conclusion

In this registry-based study, more patients were presented in MDTMs over time, but higher age at diagnosis or poorer prognosis were associated with less presentation. Presentation in MDTMs was associated with higher quality of care in univariate analysis, and better survival independently of age, comorbidities, metastatic status and cancer localisation, even after excluding patients dying within 3 months after diagnosis. There is still room for improvement of MDTMs, with a more systematic presentation of patients. The help of geriatric oncology and palliative experts could be of importance, especially when considering that MDTMs could also improve care management and quality of life in the end-of-life, but more studies are needed to confirm it.

## Supplementary Information

**Additional file 1.S1.** Factors associated with chemotherapy in metastatic patients.
**S2.** Factors associated with chemotherapy in non-metastatic N+ patients.
**S3.** Factors associated with pre-operative chemoradiotherapy in locally advanced rectal cancer patients

## Data Availability

French law does not allow researchers to share registries data which include individual-level features. Data can be accessed by researchers through application to the CNIL (*Commission nationale de l’informatique et des libertés*).
