# Supplementary material for: Multidisciplinary team meetings: are all patients presented and does it impact quality of care and survival – a registry-based study
Source: BMC Health Serv Res. 2021 Oct 1;21:1032. doi: 10.1186/s12913-021-07022-x (PMC8485542; doi:10.1186/s12913-021-07022-x)
Supplement: Supplementary file 1 — Additional file 1.S1. Factors associated with chemotherapy in metastatic patients. S2. Factors associated with chemotherapy in non-metastatic N+ patients. S3. Factors associated with pre-operative chemoradiotherapy in locally advanced rectal cancer patients [file 12913_2021_7022_MOESM1_ESM.docx]

S1. Factors associated with chemotherapy in metastatic patients

| Metastatic | Overall  n=1,090 | | Colon  n=816 | | Rectal  n=274 | |
| --- | --- | --- | --- | --- | --- | --- |
|  | *No* | *Yes* | *No* | *Yes* | *No* | *Yes* |
|  | 374 (34.3) | 716 (65.7) | 306 (37.5) | 510 (62.5) | 68 (24.8) | 206 (75.2) |
| Sex |  | ***p<0.001*** |  | ***p=0.04*** |  | ***p=0.006*** |
| Male | 177 (29.9) | 415 (70.1) | 146 (34.1) | 282 (65.9) | 31 (18.9) | 133 (81.1) |
| Female | 197 (39.6) | 303 (60.4) | 160 (41.2) | 228 (58.8) | 37 (33.6) | 73 (66.4) |
| Death within 3 months after diagnosis |  | ***p<0.001*** |  | ***p<0.001*** |  | ***p<0.001*** |
| No | 218 (24.2) | 685 (75.8) | 177 (26.6) | 488 (73.4) | 41 (17.2) | 197 (82.8) |
| Yes | 156 (83.4) | 31 (16.6) | 129 (85.4) | 22 (14.6) | 27 (75) | 9 (25) |
| Charlson Comorbidity Index |  | ***p<0.001*** |  | ***p<0.001*** |  | p =0.06 |
| 0 | 140 (25.6) | 408 (74.4) | 114 (28.2) | 291 (71.8) | 26 (18.2) | 117 (81.8) |
| 1 | 99 (42.9) | 132 (57.1) | 82 (46.6) | 94 (53.4) | 17 (30.9) | 38 (69.1) |
| >1 | 90 (43.9) | 115 (56.1) | 74 (47.4) | 82 (52.6) | 16 (32.7) | 33 (67.3) |
| Unknown | 45 (42.5) | 61 (57.5) | 36 (45.6) | 43 (54.4) | 9 (33.3) | 18 (66.7) |
| Age at diagnosis (years) |  | ***p<0.001*** |  | ***p<0.001*** |  | ***p<0.001*** |
| ≤50 | 4 (4.8) | 80 (95.2) | 4 (6.2) | 61 (93.8) | 0 | 19 (100) |
| [51-65] | 31 (11) | 251 (89) | 23 (11.8) | 172 (88.2) | 9 (9.2) | 79 (90.8) |
| [66-75] | 60 (20.5) | 233 (79.5) | 46 (21.2) | 171 (78.8) | 14 (18.4) | 62 (81.6) |
| [76-80] | 48 (38.4) | 77 (61.6) | 34 (37.4) | 57 (62.6) | 14 (41.2) | 20 (58.8) |
| >80 | 231 (75.5) | 75 (24.5) | 199 (80.2) | 49 (16.8) | 32 (55.2) | 26 (44.8) |
| Travel time quintiles (min) |  | p=0.26 |  | p=0.06 |  | p=0.74 |
| Q1 [3-10] | 68 (31.9) | 145 (68.1) | 54 (34.6) | 102 (65.4) | 14 (24.6) | 43 (75.4) |
| Q2 [11-17] | 64 (29.9) | 150 (70.1) | 46 (29.7) | 109 (70.3) | 18 (30.5) | 41 (69.5) |
| Q3 [18-28] | 69 (33.3) | 138 (66.7) | 58 (36.9) | 99 (63.1) | 11 (22) | 39 (78) |
| Q4 [29-39] | 86 (37.1) | 146 (62.9) | 72 (40.2) | 107 (59.8) | 14 (26.4) | 39 (73.6) |
| Q5 [40-75] | 87 (38.8) | 137 (61.2) | 76 (45) | 93 (55) | 11 (20) | 44 (80) |
| European Deprivation Index quintiles |  | p=0.06 |  | p=0.184 |  | ***p=0.03*** |
| Q1 (less deprived) | 65 (29.6) | 155 (70.4) | 56 (33.1) | 113 (66.9) | 9 (17.7) | 42 (82.3) |
| Q2 | 67 (31.3) | 147 (68.7) | 56 (36.8) | 96 (63.2) | 11 (17.7) | 51 (82.3) |
| Q3 | 75 (34.9) | 140 (65.1) | 58 (34.3) | 111 (65.7) | 17 (37) | 29 (63) |
| Q4 | 81 (34) | 157 (65) | 70 (38.7) | 111 (61.3) | 11 (19.3) | 46 (80.7) |
| Q5 (most deprived) | 86 (42.4) | 117 (57.6) | 66 (45.5) | 79 (54.5) | 20 (34.5) | 38 (65.5) |
| Period |  | p=0.24 |  | p=0.22 |  | p=0.77 |
| [2005-2006] | 58 (29.3) | 140 (70.7) | 46 (31.3) | 101 (68.7) | 12 (23.5) | 39 (76.5) |
| [2007-2009] | 116 (34.5) | 220 (65.5) | 98 (38.3) | 158 (61.7) | 18 (22.5) | 62 (77.5) |
| [2010-1014] | 200 (36.0) | 356 (64.0) | 162 (39.2) | 251 (60.8) | 38 (26.6) | 105 (73.4) |

.

S2. Factors associated with chemotherapy in non-metastatic N+ patients

| Non-metastatic N+ | Overall  n=842 | | Colon  n=636 | | Rectal  n=206 | |
| --- | --- | --- | --- | --- | --- | --- |
|  | *No* | *Yes* | *No* | *Yes* | *No* | *Yes* |
|  | 273 (32.4) | 569 (67.6) | 238 (37.4) | 398 (62.6) | 35 (17) | 171 (83) |
| Sex |  | ***p<0.001*** |  | ***p=0.001*** |  | p=0.97 |
| Male | 117 (27) | 316 (73) | 96 (31) | 214 (69) | 21 (17.1) | 102 (82.9) |
| Female | 156 (38.1) | 253 (61.9) | 142 (43.6) | 184 (56.4) | 14 (16.9) | 69 (83.1) |
| Death within 3 months after diagnosis |  | ***p<0.001*** |  | ***p<0.001*** |  | ***p<0.001*** |
| No | 241 (29.8) | 569 (70.2) | 210 (34.5) | 398 (65.5) | 31 (15.4) | 171 (84.6) |
| Yes | 32 (100) | 0 | 28 (100) | 0 | 4 (100) | 0 |
| Charlson Comorbidity Index |  | ***p<0.001*** |  | ***p<0.001*** |  | ***p<0.001**** |
| 0 | 90 (20.9) | 340 (79.1) | 81 (26.3) | 227 (73.7) | 9 (7.4) | 113 (92.6) |
| 1 | 78 (43.8) | 100 (56.2) | 65 (46.4) | 75 (53.6) | 13 (34.2) | 25 (65.8) |
| >1 | 87 (53.4) | 76 (46.6) | 75 (57.3) | 56 (42.7) | 12 (37.5) | 20 (62.5) |
| Unknown | 18 (25.4) | 53 (74.6) | 17 (29.8) | 40 (70.2) | 1 (7.1) | 13 (92.9) |
| Age at diagnosis (years) |  | ***p<0.001*** |  | ***p<0.001*** |  | ***p<0.001**** |
| ≤50 | 2 (3.7) | 52 (96.3) | 1 (2.6) | 37 (97.4) | 1 (6.2) | 15 (93.8) |
| [51-65] | 12 (5.1) | 223 (94.9) | 10 (6.1) | 153 (93.9) | 2 (2.8) | 70 (97.2) |
| [66-75] | 32 (14.6) | 188 (85.4) | 28 (17.5) | 132 (82.5) | 4 (6.7) | 56 (93.3) |
| [76-80] | 40 (39.2) | 62 (60.8) | 33 (43.4) | 43 (56.6) | 7 (26.9) | 19 (73.1) |
| >80 | 187 (80.9) | 44 (19.1) | 166 (83.4) | 33 (16.6) | 21 (65.6) | 11 (34.4) |
| Travel time quintiles (min) |  | ***p=0.02*** |  | p=0.126 |  | p=0.106 |
| Q1 [3-10] | 59 (34.9) | 110 (65.1) | 53 (38.7) | 84 (61.3) | 6 (18.8) | 26 (81.2) |
| Q2 [11-17] | 37 (21.9) | 132 (78.1) | 34 (27.2) | 91 (72.8) | 3 (6.8) | 41 (93.2) |
| Q3 [18-28] | 67 (36) | 119 (64) | 55 (39.9) | 83 (60.1) | 12 (25) | 36 (75) |
| Q4 [29-39] | 60 (36.6) | 104 (63.4) | 50 (41.7) | 70 (58.3) | 10 (22.7) | 34 (77.3) |
| Q5 [40-75] | 50 (32.5) | 104 (67.5) | 46 (39.7) | 70 (60.3) | 4 (10.5) | 34 (89.5) |
| European Deprivation Index quintiles |  | ***p=0.02*** |  | ***p=0.016*** |  | p=0.037 |
| Q1 (less deprived) | 33 (20.9) | 125 (79.1) | 30 (26.1) | 85 (73.9) | 3 (7) | 40 (93) |
| Q2 | 54 (30.5) | 123 (69.5) | 48 (37.5) | 80 (62.5) | 6 (12.2) | 43 (87.8) |
| Q3 | 52 (32.5) | 108 (67.5) | 46 (36.8) | 79 (63.2) | 6 (17.1) | 29 (82.9) |
| Q4 | 65 (36.6) | 114 (63.7) | 52 (37.7) | 86 (62.3) | 13 (31.7) | 28 (68.3) |
| Q5 (most deprived) | 69 (41.1) | 99 (58.9) | 62 (47.7) | 68 (52.3) | 7 (18.4) | 31 (81.6) |
| Period |  | p=0.83 |  | p=0.62 |  | p=0.71 |
| [2005-2006] | 51 (30.5) | 116 (69.5) | 42 (34.2) | 81 (65.8) | 9 (20.5) | 35 (79.5) |
| [2007-2009] | 78 (32.4) | 163 (67.6) | 68 (36.8) | 117 (63.2) | 10 (17.9) | 46 (82.1) |
| [2010-1014] | 114 (33.2) | 290 (66.8) | 128 (39) | 200 (61) | 16 (15.1) | 90 (84.9) |

S3. Factors associated with pre-operative chemoradiotherapy in locally advanced rectal cancer patients

| Locally advanced | Rectal  n=259 | |
| --- | --- | --- |
|  | Yes  70 (27) | No  189 (73) |
| Sex |  | p=0.53 |
| Male | 43 (25.8) | 124 (74.2) |
| Female | 27 (29.2) | 65 (70.7) |
| Death within 3 months after diagnosis |  | **p<0.001*** |
| No | 65 (25.6) | 189 (74.4) |
| Yes | 5 (100) | 0 |
| Charlson Comorbidity Index |  | **p=0.003*** |
| 0 | 30 (19) | 128 (81) |
| 1 | 17 (37) | 29 (63) |
| >1 | 16 (41) | 23 (59) |
| Unknown | 7 (43.8) | 9 (56.2) |
| Age at diagnosis (years) |  | **p<0.001** |
| ≤50 | 3 (14.3) | 18 (85.7) |
| [51 - 65] | 23 (23.2) | 76 (76.8) |
| [66 - 75] | 13 (17.8) | 60 (82.2) |
| [76 - 80] | 10 (31.3) | 22 (68.7) |
| >80 | 21 (61.8) | 13 (38.2) |
| Travel time quintiles (min) |  | p=0.36 |
| Q1 [3-10] | 9 (20) | 36 (80) |
| Q2 [11-17] | 12 (21.4) | 44 (78.6) |
| Q3 [18-28] | 15 (26.3) | 42 (73.7) |
| Q4 [29-39] | 18 (32.7) | 37 (67.3) |
| Q5 [40-75] | 16 (34.8) | 30 (65.2) |
| European Deprivation Index quintiles |  | p=0.08 |
| Q1 (less deprived) | 9 (14.1) | 55 (85.9) |
| Q2 | 19 (30.7) | 43 (69.3) |
| Q3 | 17 (36.2) | 30 (63.8) |
| Q4 | 11 (26.8) | 30 (73.2) |
| Q5 (most deprived) | 14 (31.1) | 31 (68.9) |
| Period |  | p=0.06 |
| [2005-2006] | 12 (37.5) | 20 (62.5) |
| [2007-2009] | 19 (35.9) | 34 (64.1) |
| [2010-1014] | 29 (22.4) | 135 (77.6) |
